# Estimating Abundance of Siberian Roe Deer Using Fecal-DNA Capture-Mark-Recapture in Northeast China

**DOI:** 10.3390/ani10071135

**Published:** 2020-07-03

**Authors:** Yuehui Li, Nana Li, Long Chen, Yueyuan Li, Zaiping Xiong, Yuanman Hu

**Affiliations:** 1 CAS Key Laboratory of Forest Ecology and Management, Institute of Applied Ecology, Chinese Academy of Sciences, 72 Wenhua Road, Shenhe District, Shenyang 110016, China; landscape@iae.ac.cn (L.C.); liyueyuanlyy@163.com (Y.L.); zaipingx@iae.ac.cn (Z.X.); huym@iae.ac.cn (Y.H.); 2 School of Biological and Chemical Sciences, Queen Mary, University of London, Mile End Road, London E1 4NS, UK; nana.lihw0208@novogene.com; 3 College of Resources and Environment, University of Chinese Academy of Sciences, Beijing 100049, China; 4 E’erguna Wetland Long-term Research Station, E’erguna City 022250, China

**Keywords:** abundance, density, *Capreolus pygargus*, Siberian roe deer, capture-mark-recapture, fecal pellets, DNA, microsatellite, forest

## Abstract

**Simple Summary:**

Estimation of population abundance or density is necessary for managing deer populations. However, there is no estimation of Siberian roe deer (*Capreolus pygargus*) in the Lesser Xing’an Mountains, northeast China where the density of roe deer is much lower than that of European or North American populations. We used fecal-DNA capture-mark-recapture to estimate the abundance and density in Liangshui National Nature Reserve. We collected 422 fecal pellet groups during two sampling periods in 2016, identified them to be 77 individuals by the DNA microsatellite technique and estimated the abundance of roe deer to be 87 deer (80–112, 95% CI) using the Program CAPTURE. Density was estimated to be 2.9 deer/km^2^ (2.7–3.7, 95% CI). Our study estimated the roe deer population abundance by a feces-based capture-mark-recapture approach in northeast China, successfully demonstrating the applicability of this feces sampling method in monitoring deer populations in this area. It also contributes to the development of low-density deer population ecology and management.

**Abstract:**

It is necessary to estimate the population abundance of deer for managing their populations. However, most estimates are from high-density populations inhabiting the forests of North America or Europe; there is currently a lack of necessary knowledge regarding low-density deer populations in different forest habitats. In this article, we used fecal DNA based on the capture-mark-recapture method to estimate the population abundance of Siberian roe deer (*Capreolus pygargus*) in Liangshui National Nature Reserve in the Lesser Xing’an Mountains, northeast China, where the deer population was found to be of a low density by limited studies. We used a robust survey design to collect 422 fecal pellet groups in 2016 and extracted DNA from those samples, generating 265 different genotypes; we thus identified 77 deer individuals based on six microsatellite markers (Roe1, Roe8, Roe9, BM757, MB25 and OarFCB304). With capture and recapture records of these 77 individuals, the abundance of roe deer was estimated to be 87 deer (80–112, 95% CI) using the Program CAPTURE. Using an effective sampling area which resulted from the mean maximum recapture distance (MMRD), we converted the population abundance to a density of 2.9 deer/km^2^ (2.7–3.7, 95% CI). Our study estimated the roe deer population abundance by a feces-based capture-mark-recapture approach in northeast China, successfully demonstrating the applicability of non-invasive genetic sampling in monitoring populations of deer in this area, which contributes to the development of low-density deer population ecology and management.

## 1. Introduction

Estimating population abundance or density is one of the basic requirements for wildlife research because it is important to understand a population’s structure and dynamics and how that population reacts to the environment [1,2]. For example, estimates of vital rates and instantaneous population growth rates must be interpreted in the context of density or relative abundance [3]. Meanwhile, population management requires an accurate estimate of population abundance or density along with other population parameters to identify management goals, such as the balance between population abundance and resource capacity [4]. Historically, estimating animal population abundance or density, especially for Cervids, which are globally widespread, has been explored extensively. However, most existing studies have been conducted in areas with a high-density deer population [5,6]. For example, it is common to see a deer density in excess of 10 deer/km^2^—even up to more than 25 deer/km^2^—in the United States or Europe [7,8]. Consequently, in those areas, deer abundances, even sex-specific or habitat-specific abundance, have been intensively monitored by various methods in order to facilitate deer management, limiting or controlling their population size within bounds [4,9]. In contrast, very little research has been conducted in low-density deer population areas where the population abundance is urgently required for effective management [10]. For example, in northeast China, the limited investigation data show that the Siberian roe deer (*Capreolus pygargus*) density commonly appears to be <1 deer/km^2^ (according to forest animal resources surveys in Heilongjiang province, 2001) [11,12]. More studies are needed to support the roe deer restoration regionally.

In order to monitor the population abundance, instead of the fecal pellet-group count method which remains contentious due to its evaluation across spatial and temporal scales and infrequently quantified precision despite its wide use for over half-century, the non-invasive genetic capture-mark-recapture (CMR) method has been proven to produce the most accurate and the least observer-biased estimation for mammals, especially in densely vegetated environments where direct observation or capture is difficult or impossible [13,14,15,16,17,18,19,20]. This molecular technology has sparked a revolution in wildlife monitoring by identifying individuals using DNA from hair, tissue and scat samples, without directly observing or capturing individuals in the field. Especially, because fecal DNA can be obtained easily, over the past 10 years, fecal-DNA CMR has been widely used since its first successful application in coyotes (*Canis latrans*), followed by studies conducted on Cervidae including red deer (*Cervus elaphus*) [21], Sitka black-tailed deer (*Odocoileus hemionus sitkensis*) [19,22], roe deer (*Capreolus capreolus*) [23], woodland caribou (*Rangifer tarandus caribou*) [24], white-tailed deer (*Odocoileus virginianus*) [25], Columbian black-tailed deer (*Odocoileus hemionus columbianus*) [3] and mule deer (*Odocoileus hemionus*) [26]. Most of those studies were conducted in European or American study areas [19], developing a robust protocol for identifying the field and laboratory sampling procedures of fecal-DNA CMR and enhancing greatly the precision of population abundance estimation compared with the pellet-group count method there. However, few studies were reported about the protocols and the applicability of non-invasive genetic sampling for low-density deer populations.

The abundance of low-density deer populations needs to be accurately and precisely estimated in the context of a robust design survey plan and for genetic protocols in Northeast China. Most studies in northeast China have used the more indirect methods of the footprint trail count [27], sign count [11] or image count from camera traps [28] rather than pellet-group counts, yielding low precision and an unquantifiable sampling bias for abundance estimation, because those procedures—which convert signs or foot trails to individual count—are not reliable due to the signs or foot trails left by unknown individuals [29,30]. Only one study there has used fecal DNA to identify individual red deer (*Cervus elaphus*) to estimate their abundance; however, this study did not perform recapture, and thus contributes little to the development of a robust sampling design and protocols [31]. Those limited existing estimates are not enough to reveal the comparative abundance or density of the population and therefore cannot support a further understanding of population abundance-related issues. Therefore, using fecal-DNA capture-mark-recapture to estimate the roe deer population abundance is necessary and suitable—although challenging—in northeast China. Our objectives were to identify the effectiveness of genetic sampling from fecal pellets and estimate the roe deer population abundance and density using the capture-mark-recapture method in Liangshui National Nature Reserve, located in northeast China.

## 2. Materials and Methods

### 2.1. Study Area

**Figure 1:**
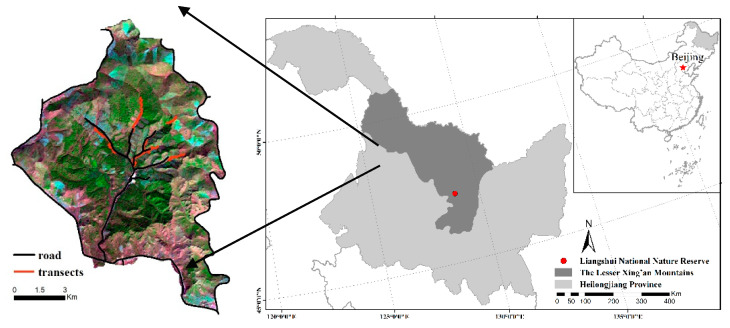
The study area located in the Lesser Xing’an Mountains, northeast China and transect pattern.

Our study was conducted in Liangshui National Nature Reserve (128°47′8′′~128°57′19′′ E, 47°6′49′′~47°16′10′′ N) covering 121.33 km^2^ in the Lesser Xing’an Mountains, northeast China (Figure 1). The study area has been strictly protected from human activity since it was established as a provincial reserve in 1980 and further upgraded to a national reserve in 1997 [32]. The study area is characterized by rolling mountainous terrain, with an altitude ranging from 350 to 700 m above sea level and an average slope gradient of 10°~15°. The mean annual temperature was −0.3 °C, with a mean temperature of 7.5 °C in August and −6.6 °C in January. The mean annual precipitation was 676 mm concentrated in June, July and August. The forest was primarily dominated by Korean pine (*Pinus koraiensis*) mixed with other woody species such as Korean spruce (*Picea koraiensis*), Khingan fir (*Abies nephrolepis*), mono maple (*Acer mono*), dahurian larch (*Larix gmelinii*) and Manchurian walnut (*Juglans mandshurica*), a variety of shrubs such as honeysuckle (*Lonicera chrysantha*), hazelnut (*Corylus mandshurica*), false spirea (*Sorbaria sorbifolia*), spiraea; (*Spiraea salicifolia*) and grasses such as cotton grass (*Carexcal-litrichos*), sedge (*Carex campylorhina*) and Meadow pine (*Equisetum silvaticum*) [33,34,35]. Data derived from camera-trapping indicated that roe deer are the only common mid-size wild ungulate species in the Reserve currently [36], with no other animals producing similar fecal pellets. No other natural carnivore predators were found to dwell in the area [36].

### 2.2. Sampling Design 

**Figure 2:**
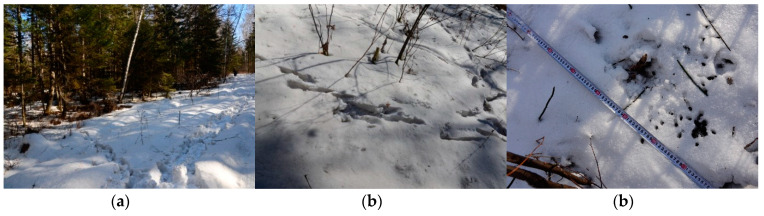
The point where we encountered the newly left deer trail (**a**). The newly left deer trails which were fresh, maintained their shape and were identifiable. (**b**), and the pellets sampled on the trails, which were black, round and moist (**c**).

We first sampled roe deer fecal pellets from 15 to 21 March 2016. The survey team included five members, of which one was a local guide, one searched for fresh pellets, another two recorded and collected the pellets and one cleared the remaining pellets and labelled transects. We walked along the forest road until we encountered the first newly left deer trail, which was easily distinguished from old trails, which were dusty, reshaped and even unidentifiable due to wind and rain. This point was considered as starting point (Figure 2a,b). We would then survey this trail for a 5 m width transect at each side from the transect centre to collect fecal pellets until we encountered other newly left trails. If other trails intersected with the trail being surveyed, we continued with the trail which was most parallel with the direction of the road as determined by the compass imbedded in GPS (Trimble Company, Sunnyvale, CA, USA). We tied red cloth strips on trees to mark transects at 10–20 m intervals depending on tree density; meanwhile, we also recorded the trail with GPS, ensuring repeatability across field seasons [19].

When we encountered a fresh fecal pellet group which appeared to be black, round and moist along transect (Figure 2c), we used sterile gloves and tweezers to collect approximately 10–15 fecal pellets and stored them in paper envelopes labelled with coordinates and dates. Then, we put them in a plastic box temporarily, and then in a vehicle refrigerator about 1–2 h after collection. If the pellets were along a single footprint line which was obviously deposited by the same individual during movement, one sample portion was collected from the group. After sampling, we removed all remaining pellets within 5 m of each side from the trail centre to make sure no old pellets would be mixed in the next sampling occasion. The samples were taken back to the laboratory about 4–5 h after collection and stored at −20 °C in the freezer until DNA extractions. 

Following this method, five transects were finally used for sampling, for which the straight-line distances average 1.68 km, and each transect was at least 1.60 km distant from others. The real roe deer foot trails (including running around as well as to and fro) in transects were found to count for 5.12 km on average. There is no estimation of home range size of roe deer in our study area and close areas. We referenced the European roe deer (*Capreolus Capreolus*) home range size ranging from 0.22–0.51 km resulted from those areas within 46°~48° N(similar to our study area) in France and Italy [37,38,39,40]. Thus, the movement radius ranges from 0.26 to 0.40 km.

We repeated the sampling route marked by red cloth strips to collect fecal pellets from 20 to 27 December in 2016. When we encountered newly left feces along transects marked by a red cloth strip and GIS, we sampled, transported and stored them by the same method [19,41,42].

### 2.3. Genetic Analysis

#### 2.3.1. DNA Extraction 

Microsatellite analysis was conducted by Microread Company (Beijing, China) within 4–6 weeks after field selection. Fifteen groups of fecal pellets were randomly selected to preliminarily assess candidate microsatellite markers. The QIAamp DNA Stool Mini Kit (Magen, Beijing, China) was used to extract fecal DNA following the manufacturer’s instructions. The success of the DNA extraction and the purity of genomic DNA were examined in 1.2% agarose gel electrophoresis using 2 μL DNA. The DNA was stored at −20 °C.

#### 2.3.2. Microsatellite Marker Selection 

We selected the 18 most used loci in previous publications from European roe deer (*Capreolus capreolus*) as candidate microsatellite markers (Table A1), in which IDVGA58, MB25 and BM757 were also used in Siberian roe deer individual identification [43]. All the microsatellite markers were amplified as single reactions; the standard cycling and buffer conditions for the polymerase chain reaction (PCR) were as follows: predenaturation for 5 min at 95 °C; 25 cycles of 94 °C denaturation for 30 s, 56 °C annealing for 30 s, 72 °C extension for 30 s; 15 cycles of 94 °C denaturation for 30 s, 53 °C annealing for 30 s, 72 °C extension for 30 s; and a final extension at 60 °C for 30 min. All PCR products were stored at 4 °C. The polymerase chain reaction (PCR) was conducted using a Taraka Taq PCR kit (Takara Biomedical Technology, Beijing, China). The total PCR system volume was 15 μL, including 1.5 μL 10 × BufferI, 1.2 μL deoxynucleoside triphosphate (dNTP) (2.5 mM), 0.8 μL TP-M13 (5 μM), 1 μL each of upstream and downstream primers (5 μM), 0.1 μL Takara HS Taq(5μ/μL), 1.2 μL template DNA, and sterilized deionized water to a total volume of 15 μL. 

The PCR products were genotyped using ABI 3730XL (Applied Biosystems, Foster City, CA, USA), and allele sizes were estimated using Genemapper 4.0 (Applied Biosystems, Foster City, CA, USA) and scored by visual examination. Using samples from 15 pellet groups, we excluded markers that amplified weakly or failed to amplify [18]. 

Candidate markers were also evaluated for their performance in terms of the probability of identity (PID), polymorphism information content (PIC) and allele heterozygosity(H) [44]. PID is the probability that two random individuals drawn from a given population share identical genotypes at all genotyped loci [45]. This value defines the upper limit of the possible ranges for the probability of identity in a population, suggesting the most conservative number of loci that were required to recognize all individuals in the population. The PID and PID_sibs_ (PID among siblings)values were computed using GIMLET version 1.3.3 [44,46] (http://pbil.univ-lyon1.fr/software/Gimlet/gimlet.htm), both with the acceptance value of <0.01 [47,48,49]. The PIC was conducted in Cervus 3.0 (Field Genetics, London, UK) according to the assessment standards that when PIC > 0.5, the locus was considered to have a high polymorphism information content; if 0.25 ≤ PIC ≤ 0.5, an intermediate polymorphism information content was assumed; and if PIC ≤ 0.25, the locus was considered to have a low polymorphism information content. The number of alleles (A), observed heterozygosity (H_O_), expected heterozygosity (H_E_) and effective number of alleles (A_E_ = 1/(1−H_E_)) were also calculated using Cervus 3.0. Six markers were selected for the roe deer individual identification in our study, which were Roe1, Roe8, Roe9, BM757, MB25 and OarFCB304.

#### 2.3.3. Genotyping and Individual Identification

The six selected loci were amplified in one multiplex PCR (polymerase chain reactions) using the same thermal cycling conditions as the previous single reactions. The PCR was completed without a certain number of repetitions because the experiment protocols are well documented in previous studies [50]. The samples that failed to amplify at any of 6 loci in PCR had 2–3 more rounds of DNA extraction; if a sample failed three times, it was be considered as an unqualified sample. The genotypes of successful samples were matched using the Numerical Taxonomy Multivariate Analysis System software package (NTSYS-pc 2.0, Exeter Software, Setauket, NY, USA) [51].

### 2.4. Population Abundance Estimation

We used the Program CAPTURE based on the capture-mark-recapture method [52] in the Program Mark [53] to estimate the population abundance. 

Program CAPTURE incorporates time (*t*), behavioural (*b*) variation, and individual heterogeneity (*h*) in the estimation of capture and recapture probabilities in a likelihood framework. A suite of eight models can be used, including M*_o_* (null model with all capture and recapture probabilities equal), M*_t_*, M*_b_*, M*_h_*, M*_th_*, M*_tb_*, M*_bh_* and M*_tbh_* [54].

Based on the results of individual identification using the six microsatellite markers from the two surveys, the capture history record file was created for each sample, where “1” stands for being captured and “0” stands for not being captured. If the same genotype was detected in more than one sample, only one sample was recorded [52]. Therefore, the capture history for each individual fell into the categories “01”, “10” or “11”. A range of models were tested, and their fitting values (0–1) were given by the Chi-square goodness–of-fit test built in the Program CAPTURE; then, the best-fitting model with the highest fitting value was chosen for population estimation. 

### 2.5. Population Density Estimation

Knowing the abundance of roe deer, the estimated density ($\hat{D}$) was further calculated by dividing abundance estimate ($\hat{N}$) using the effective sampling area ($\hat{A}$) (i.e., $\hat{D}$ = $\hat{N}$/$\hat{A}$). The effective sampling area is determined by the mean maximum recapture distance (MMRD) which has been found to be the most conventional and accurate method to delineate the area and has been used in several species of mammals [19,26].First, the distances between any two samples of a recaptured individual were calculated, and the largest number was counted as the maximum distance of one individual movement. Then, the MMRD was obtained from the median value among individual maximum distances of all recaptured roe deer. Finally, we assigned a strip boundary around each transect (originally 5 m wide) by creating a buffer with a width equal to the MMRD and the area within the strip boundary was the effective sampling area ($\hat{A}$) in the equation [3].

## 3. Results

### 3.1. Fecal Sampling and Genotyping

Analyses of the heterozygosity of the six selected markers Roe1, Roe8, Roe9, BM757, MB25 and OarFCB304 are presented in Table 1. From all the samples genotyped, a total of 64 alleles were detected with lengths ranging from 62–212 bp, with 10.7 alleles and 4.167 effective alleles at each locus on average. The locus OarFCB304 was detected to have the most alleles, at 19, and MB25 had the least with three; other loci were detected to have 4–16 alleles. The observed heterozygosity (H_O_) was between 0.192–0.909 with a mean value of 0.624; the expected heterozygosity (H_E_) was between 0.183–0.866 with a mean value of 0.649. The PIC of the six markers ranged from 0.175 to 0.899, in which Roe8, BM757 and OarFCB304 were high PIC loci; Roe9 and MB25 were intermediate PIC loci; and only Roe1 was a low PIC locus. The six markers together have a high PIC. 

In two surveys, we collected 422 fecal pellet groups and successfully genotyped 265 (62.80%) pellet groups, identifying 77 individuals with a genetic similarity coefficient of 0.95 (Table 2).

### 3.2. Population Abundance Estimation 

The capture history file was generated based on the identification of the 77 individuals; 43 individuals were recorded as “10”, 21 individuals as “01” and 9 individuals as “11”. The models tested with their increasing fitting values were as follows (Table 3): M*_tbh_* (1.00), M*_bh_* (0.86), M*_o_* (0.73), M*_t_* (0.33), M*_th_* (0.14), M*_b_* (0.08), M*_tb_* (0.11) and M*_h_* (0.00). Therefore, the best-fitting model M*_tbh_* was used to estimate the population abundance, which was 87 deer (80–112 deer within the 95% confidence intervals) (CI).

### 3.3. Population Density Estimation

We estimated an MMRD of 814 ± 626 m (n = 39) based on all individuals sampled more than once in the study. Our effective sampling area for roe deer was estimated to be 29.95 km^2^. Finally, we used this effective area to convert abundance estimates to density, resulting in 2.9 deer/km^2^ and 2.7~3.7 deer/km^2^ being within the 95% confidence interval.

## 4. Discussion

### 4.1. Sampling Design and Genetic Analysis 

Our study suggests that the fecal-DNA capture-mark-recapture method is effective in the low-density roe deer population area, providing a feasible and efficient sampling design and genetic analysis. The feasibility and efficiency of this method are facilitated by the high-encounter rates with feces [19]. One of the challenges of sampling low density deer is to get enough captures and recaptures for the effective CMR analyses. Generally, in order to capture or recapture feces, two types of field designs for sampling feces have been developed: one is designed as systematic transects covering a certain unit area, which has been used widely in the USA and Europe where the deer density is commonly high. For example, Ebert (2012) sampled feces of black-tailed deer along 20 transects which were on average 5.7 km in length and 0.32 km in distance between transects in a study area of approximately 40 km^2^ in southwest Germany [23]. This method seems to be only effective in studying high deer-density populations, whereas in a low-density population, such as in a pilot study conducted in other areas close to our study area, we failed to directly count either deer individuals or fecal pellets groups following the systematic transects because the samples collected were insufficient for statistical analysis (unpublished data). The other method is to trace and sample the footprint trails newly left by deer to establish transects, as we have used in our study, which was first developed by Brinkman and applied successfully in Alaska, USA [19]. The latter design has been shown to increase the encounter probability greatly, especially in lower deer-density or/and dense forest [55]. Providing enough opportunities to obtain 422 samples in two surveys, this design is proven to be appropriate and essential in our study area. 

In addition, the feasibility and efficiency of this method are related to the genotyping success of samples. According to other genotyping for abundance estimation such as 65% produced by Hedmark et al. (2004) [16], 74% produced by Kendall et al. (2008) [56] and 44%, 45% and 87% produced by Brinkman et al. (2011) [19], our genotyping success rates were reasonable. Uncertainty of genotyping success rates were mainly affected by the sampling time, storage process and loci selected for individual identification, as described below.

First, the sampling time significantly affected the genotyping success rate of samples. The sample genotyping success rates were found to be 52.5% in March and 78.8% in December. The difference mainly resulted from the feces quality, for example, degradation after deposition is found to increase with increasing rainfall and temperature [22,57,58].In a study on white-tailed deer [25], individual identification using fecal DNA, amplification success rates were 28% and 79% before and after the rain event, respectively. In our study, in December with a temperature range of −25 °C to −11 °C, cold and dry weather helped: the DNA amplification success was 78.8% for samples, the feces has been frozen since it is deposited. However, in March when the peak temperature occasionally reached 5 °C and the snow was melting in the afternoon, the DNA amplification success lowered to 52.5% for samples. Additionally, we also collected samples after the snow melted in April 2016 (temperature on sampling days ranged −1.3–8.3 °C), but most samples failed to amplify. Roy and colleagues [59] have revealed that moose feces should be collected during late winter and very early spring before the weather is getting warm in April. Given that their study area in the John Prince research forest (54°40′10.1′′N~124°24′52.1′′E) which is 10° north away from our study area, we suggest that the best season for sampling roe deer feces in northeast China is in or before March.

Second, the sample storage process can impact the amplification success rate negatively if it fails to prevent the decomposition of the DNA on the surface of feces before extraction. In most studies, feces were stored frozen [23,24] or in 95–100% ethanol [3,19] and others were kept dry [25], with a genotyping success rate in the range of 49.3–93%. In previous feces sample survey studies in China, samples were mostly preserved frozen with high success rates in the range of 79.5–98.5%; this was therefore considered to be the best storing method for roe deer feces samples in the field [31,60,61]. Kept frozen, our DNA amplification success rate averages 62.8%, which is intermediate, and the samples collected in December alone had a higher success rate at 78.8%, suggesting that this is a robust way to collect, keep and deliver feces in winter, especially in northeast China. 

Third, the characteristics of the selected loci impact the genotyping success. Due to the lack of previous work using the microsatellite technique for roe deer in northeast China, we considered 18 candidate loci from European roe deer or Siberian roe deer, and six of them were finally used to identify the individuals due to their success in DNA amplification and good performance in allele numbers, PIC, PID and PID_sib_. Overall, six markers were detected with multiple alleles, and together they were sufficient to discriminate individuals from each other reliably, even for closely related siblings as shown by the PID_sib_ (being much lower than 0.01) (Table 1). Of the six selected loci, four were used to identify European roe deer individuals, and they worked well for identifying Siberian roe deer. Therefore, our research further suggests that the same loci can be used for close species and even species in close families, allowing the DNA individual identification to become more practical and more widely used. 

### 4.2. Population Abundance Model

We used the Program CAPTURE to estimate the abundance, ignoring population dynamics because our study population is suggested to be assumed to be geographically and demographically closed. We suggest it is reasonable to ignore emigration and immigration because the study area was located in an upper watershed and protected from human activities occurring around this area, which is always considered to be a relatively closed area for deer. We also ignored birth and death. First, some percentage of mortality usually can be caused by a severe winter, predation and hunting activities [62], but between the two field occasions, summer, autumn and early winter occurred, with the temperate averaging from −6.5 °C to 0 °C, remaining at the same level as the normal one, and no natural predators were observed such as wolf and lynx in a recent camera trapping effort [36], and there should therefore not have been a significant population loss. Additionally, given that hunting is banned by law in this reserve, we can assume that mortality was low and therefore had little effect on population change. Second, deer breeding is expected in June and new births should occur; however, there is a lack of knowledge—and no reporting—regarding the birth rate of the roe deer population in our study area, meaning that this must currently be ignored and needs to be studied further. In fact, our study is in line with some other studies which have assumed that birth, death, immigration and emigration have no significant effect on estimates when using the closed population model under similar conditions [3,19,24,26]. In terms of the above model’s assumptions and pre-conditions, the Program CAPTURE is suitable for our study population in a low deer-density area in northeast China and continuous work on estimation precision will be needed to create comparable data. 

### 4.3. Population Abundance and Density

With this fecal-DNA capture-recapture method, we provide a rigorous estimate of abundance and density with high precision for roe deer in northeast China. The density was estimated to be 2.9 deer/km^2^ (2.7–3.7, 95% CI), exhibiting the same low level of density as previous studies. The density of deer was found to be generally lower than 1 deer/km^2^ in northeast China. For example, the roe deer density was <1 deer/km^2^ throughout Heilongjiang province, in which the Lesser Xing’an Mountains are located (forest animal resources surveys in Heilongjiang province, 2001); the roe deer population density was estimated at 0.50 ± 0.04 deer/km^2^ in Wanda Mountain [12] and 0.15 deer/km^2^ in Hunchun Natural Reserve [11]; and the relative abundance index (RAI, the number of samples trapped by a camera during 100 days) of roe deer was reported as 1.53, which was lower than the value of 2.76 for livestock in Hunchun National Nature Reserve [28].

As the first capture-mark-recapture population estimation for roe deer abundance, our study is different from previous population estimations in northeast China in terms of the data collection and analysis method and the area used to convert the abundance to density. In terms of the data collection and analysis method, except for Tian’s study [31], the exiting estimates were often analysed by the data from a survey of the number of roe deer footprint trails without distinguishing and identifying individuals. So far, there is a lack of strong evidence to explain the relationship between the number of footprint trails and the number of roe deer individuals. In terms of the area used to convert abundance to density, the current studies in China estimated the density by dividing the population size by the whole study area instead of the effective area [12,31,42]. In general, the effective area is always smaller than the whole study area, meaning that using the whole area is still very likely to underestimate the deer density to some degree. Furthermore, the difference of estimates between our study and previous studies causes difficulties in validating our modelling because no previous data can be used. However, testing against known numbers may be the only way to fully validate a population estimation [19], and we therefore aim to continue our research on this population after this successful start; we also suggest more roe deer abundance research using the fecal-DNA capture and recapture method on other populations, contributing to the roe deer database in northeast China.

Following the method from Brinkman’s protocol [19], our studies yielded the abundance and density of roe deer population that can be compared with estimates conducted in other countries. Our results are much lower compared to the deer abundance which resulted from estimates using a non-invasive CMR method in other forest landscapes in Europe or North America; for example, the highest observed roe deer population density reached 53.8 deer/km^2^ in Europe [63]. The wide gap is presumably due to changes in habitat which resulted from human activities such as timber harvesting. It is common that the abundance of deer has been decreasing from the 1950’s when the forest was initially timbered for commercial products in northeast China. For example, roe deer density was estimated at 2.60 deer/km^2^ in 1998 and 0.50 ± 0.04 deer/km^2^ in 2002 in Wanda Mountain due mainly to fragmentation and loss of habitat [27].Thus, the population size is speculated to be lower than a normal level appropriate to the surrounding resources and environment in forest landscapes.

Similar to most studies, we converted the population abundance to density by dividing the abundance ($\hat{N}$) by the effective area ($\hat{A}$) assessed by the mean maximum distance (MMRD). This conversion adds another level of uncertainty associated with assumptions about the effective area sampled [3]. If the extent of the sampling area is smaller than home ranges, using MMRD will underestimate the effective area and thus result in positive bias for density estimates [26,64,65,66]. Lacking data revolving home range size and exact movement radius in the study area and even throughout northeast China, we had to use the other findings resulted from study areas similar in geographical location to design transects. Either, we have no chance to use the spatial capture-recapture modelling method which was proven to directly estimate the density, decreasing the biases stemming from those incorrect assumptions about effective sampling area.

## 5. Management Implications

Our protocol, following Brinkman’s method [19], has been successful; it should therefore be reasonably accurate and robust when applied to deer monitoring in other areas in the Lesser Xing’an Mountains where the natural environment conditions are similar [67]. Meanwhile, the cost for the genetic analysis service by the Microread Company was ¥165 (approximately US $24) per sample, which was less than Goode’s US $60 per sample for the analysis of a white-tailed deer population estimation conducted in America in 2014 [25], making it much more affordable to use DNA in the routine estimation of population abundance.

The application of this method, on one hand, can provide information on demographic features and dynamics, and potential predictions combining population features such as age structure and sex ratio. Those results are necessary to aid current management measures to increase the low-density roe deer population to a desirable density or possibly to reduce the population if it is overpopulated in future. On the other hand, apart from individual identity, much more information can be obtained from pellet groups including genetic diversity, habitat use, forage preference, home range and disease [25], and such exploration from fecal samples at multiple scales would greatly improve our understanding of roe deer biology and ecology in the northeast of China. 

## 6. Conclusions

The fecal-DNA capture-mark-recapture method is found to be effective in the dense forest landscape of the Lesser Xing’an Mountains, northeast China, where the roe deer appear to be at a low density and are difficult to observe. The application of this sampling design and genetic analysis is guaranteed by the (re)capture rate and genotyping success rate of roe deer feces encountered along the trails. Apart from the loci being used, we confirmed that the genotyping success itself was significantly affected by sampling time and its storage, therefore we suggest that the best season for sampling roe deer feces in northeast China should be in or before March to avoid sample degradation and contamination. Moreover, using an effective sampling area which resulted from the mean maximum recapture distance (MMRD), we converted the population abundance result to a density of 2.9 deer/km^2^ (2.7–3.7, 95% CI), which supports the observation that the density of deer in North China is lower than that of European and North American populations. 

## Figures and Tables

**Table 1:** Genetic diversity statistics, including number of allele (A), effective number of alleles (A_E:_); expected heterozygosity (H_e_), observed heterozygosity (H_O_), polymorphism information content (PIC), unbiased probability of identity (PID), and probability of identity for siblings (PID_sibs_) for the six loci that were used to genotype the fecal DNA of Siberian roe deer in Liangshui National Nature Reserve in the Lesser Xing’an mountains, China in 2016.

Locus	A	A_E_	H_E_	H_O_	PIC	PID	PID_sibs_
Roe1	5	5	0.192	0.183	0.175	7.785 × 10^−7^	8.276 × 10^−3^
Roe8	16	4	0.909	0.879	0.866	3.790 × 10^−4^	9.560 × 10^−1^
Roe9	4	3	0.483	0.551	0.462	3.140 × 10^−6^	1.693 × 10^−2^
BM757	17	7	0.796	0.908	0.899	1.474 × 10^−2^	3.008 × 10^−1^
MB25	3	3	0.498	0.505	0.381	1.157 × 10^−6^	9.998 × 10^−3^
OarFCB304	19	6	0.868	0.870	0.855	1.088 × 10^−5^	3.092 × 10^−2^
Mean	10.7	4.167	0.624	0.649	0.606	--	--

**Table 2:** Number of Siberian roe deer fecal samples collected and genotyping success rate in two surveys in 2016 in Liangshui National Nature Reserve, China.

Date	No. of Pellet Groups Collected	Successfully Genotyped	Success Rate in Genotyping
December 2016	165	130	78.8%
March 2016	257	135	52.5%

**Table 3:** Estimated population abundance for Siberian roe deer in Program CAPTURE using the fecal-DNA capture-mark-recapture method in Liangshui National Nature Reserve, China in 2016. Results from different models in the programs are shown with fitting values in brackets where applicable.

Model (Fitting Value)	Roe Deer Abundance (Within 95% CI) (Deer)	SE
M*_tbh_* (1.00)	87 (80–112)	7
M*_o_* (0.73)	201 (134–350)	52
M*_t_* (0.33)	182 (125–311)	44
M*_tb_* (0.11)	78 (78–92)	23
M*_t_-* Chao	176 (122–295)	42
M*_b_-* Chao	334 (195–640)	107
M*_h_* (0.00)	110 (99–127)	7

## Appendix A

**Table A1:** Candidate loci used in identifying Siberian roe deer (*Capreolus pygargus*) individuals using fecal DNA to estimate population abundance in Liangshui National Nature Reserve in the Lesser Xing’an Mountains, China, in 2016.

Locus	Sequences (5′-3′)	Length (bp)	A *	References
Roe1	F AAATTTGGCTCTGCAATCGG R ACACAAAAGCCACCCAATAC	112–132	7	[68]
Roe6	F GTTCCTAGCCCAGTGCTC R TGCAGACCTGGCAGAC	89–109	3	[68]
Roe8	F AAGCCGCGCTTGAAGGAG R ATCAAGCTCCCCTCTTCG	69–89	7	[68]
Roe9	F TTGGCGTCATTCCAACAGAG R TCACAGCAGAATGTCATCTG	175–179	3	[68]
IDVGA8	F CTCTTGGGGGCGTGTTGTCT R TAGCAGAAAGCACAGGAGTC	209–225	4	[69]
BM757	F TGGAAACAATGTAAACCTGGG R TTGAGCCACCAAGGAACC	172–204	10	[69]
MB25	F GGACACGTTCTGCAGATACAACTAC R GAACTCTCCTTAAGCATACTTGCTC	198–200	2	[70]
OarFCB304	F CCCTAGGAGCTTTCAATAAAGAATCGG R CGCTGCTGTCAACTGGGTCAGGG	158–177	15	[69]
BL42	F CAAGGTCAAGTCCAAATGCC R GCATTTTTGTGTTAATTTCATGC	225–231	3	[70]
BM1818	F AGCTGGGAATATAACCAAAGG R AGTGCTTTCAAGGTCCATGC	249–267	3	[70]
BMC1009	F GCTTGGATGGACCATGTTG R CACTTGAGGGGCAAATGATT	282–290	3	[70]
BMS119	F TTAAGCAGGGACGAACGTG R AATTGCCAGGAAGATTGTGG	99–105	4	[70]
ETH225	F GATCACCTTGCCACTATTTCCT R ACATGACAGCCAGCTGCTACT	139–145	4	[70]
HUJ1177	F TCCATCAAGTATTTGAGTGCAA R ATAGCCCTACCCACTGTTTCTG	199–219	6	[69]
ILST030Q	F CTGCAGTTCTGCATATGTGG R GTTTCTTCTTAGACAACAGGGGTTTGG	157–165	3	[70]
KCNA4	F CTGGAAGAGATGTTAAAAGTA R CACTGAATAAACAACTGCTCA	220–250	3	[70]
MAF70	F CACGGAGTCACAAAGAGTCAGACC R GCAGGACTCTACGGGGCCTTTGC	139–161	5	[70]
SR-CRSP1	F TGCAAGAAGTTTTTCCAGAGC R TCATTCTCAGGAAACTCTGAAAC	127–151	5	[70]

* Number of alleles. F: Forward primer, R: Reverse primer.
